# A two-sample Mendelian randomization study explores metabolic profiling of different glycemic traits

**DOI:** 10.1038/s42003-024-05977-1

**Published:** 2024-03-08

**Authors:** Tommy H. T. Wong, Jacky M. Y. Mo, Mingqi Zhou, Jie V. Zhao, C. Mary Schooling, Baoting He, Shan Luo, Shiu Lun Au Yeung

**Affiliations:** 1 School of Public Health, Li Ka Shing Faculty of Medicine, The University of Hong Kong, Hong Kong SAR, China; 2 Department of Biological Chemistry, School of Medicine, University of California Irvine, Irvine, CA USA; 3 Center for Epigenetics and Metabolism, University of California Irvine, Irvine, CA USA; 4 School of Public Health and Health Policy, City University of New York, New York, NY USA

**Keywords:** Endocrinology, Type 2 diabetes, Diagnostic markers, Type 2 diabetes

## Abstract

We assessed the causal relation of four glycemic traits and type 2 diabetes liability with 167 metabolites using Mendelian randomization with various sensitivity analyses and a reverse Mendelian randomization analysis. We extracted instruments for fasting glucose, 2-h glucose, fasting insulin, and glycated hemoglobin from the Meta-Analyses of Glucose and Insulin-related traits Consortium (*n* = 200,622), and those for type 2 diabetes liability from a meta-analysis of multiple cohorts (148,726 cases, 965,732 controls) in Europeans. Outcome data were from summary statistics of 167 metabolites from the UK Biobank (*n* = 115,078). Fasting glucose and 2-h glucose were not associated with any metabolite. Higher glycated hemoglobin was associated with higher free cholesterol in small low-density lipoprotein. Type 2 diabetes liability and fasting insulin were inversely associated with apolipoprotein A1, total cholines, lipoprotein subfractions in high-density-lipoprotein and intermediate-density lipoproteins, and positively associated with aromatic amino acids. These findings indicate hyperglycemia-independent patterns and highlight the role of insulin in type 2 diabetes development. Further studies should evaluate these glycemic traits in type 2 diabetes diagnosis and clinical management.

## Abstract (web summary)

A Mendelian randomization study on metabolic profiling of different glycemic traits suggests hyperglycemia-independent patterns and highlights the role of insulin in the development of type 2 diabetes.

## Introduction

Hyperglycemia, increased insulin, and type 2 diabetes likely increase the risk of cardiovascular disease^1^. A recent study suggested there were different roles of four glycemic traits in atherosclerotic and thrombotic conditions^2^. Based on the American Diabetes Association “Standards of Medical Care in Diabetes”, type 2 diabetes may be diagnosed based on different glycemic traits. Diagnosis can be based on the glucose criteria (including fasting glucose, 2-h glucose after a 75-g oral glucose tolerance test) or the A1c criteria (glycated hemoglobin (HbA_1c_))^3^. However, these could lead to heterogeneity in the profiling of type 2 diabetes given these glycemic traits do not always underlie the same pathophysiology relevant to glucose homeostasis^4^. Clarifying the similarities and differences in the association with downstream metabolites (e.g., lipid subfractions and amino acids) of these glycemic traits and liability to type 2 diabetes may help clarify the pathophysiology of these inter-related glycemic traits in various diseases, such as cardiovascular diseases shown in a previous Mendelian randomization study^2^.

Previous observational studies have suggested glycemic traits are associated with particular metabolites. Studies of Finnish adults (*n* = 7098) found that branched-chain amino acids (BCAAs) and ketone body levels were associated with lower insulin sensitivity and higher risk of type 2 diabetes^5,6^. Another study of Finnish men (*n* = 9399) found that higher concentrations of very low-density lipoproteins (VLDL) subclass particles were associated with glucose intolerance and newly diagnosed type 2 diabetes^7^. A small observational study (*n* = 733) showed HbA_1c_ positively associated with some BCAAs, such as isoleucine and alanine, and lower apolipoprotein A1 (ApoA1)^8^, whilst another small study (*n* = 155) suggested possible differences in amino acid signatures for prediabetes defined by different glycemic traits^9^. Nonetheless, these associations may be susceptible to residual confounding by obesity and physical inactivity. A Mendelian randomization study, which makes use of genetic endowment randomly allocated at conception, can overcome these limitations^10^. We used a Mendelian randomization study to infer the role of each glycemic trait (fasting glucose, 2-h glucose, HbA_1c_, and fasting insulin) and liability to type 2 diabetes in metabolomic signatures with various sensitivity analyses and a reverse Mendelian randomization analysis.

## Methods

This is a two-sample Mendelian randomization study using summary statistics from genome-wide association studies (GWAS), which relies on the three instrumental variable assumptions^11^. First, the instruments should be strongly associated with the exposure of interest. Second, there should be no unmeasured confounding of instruments on outcome. Third, the instruments should be independent of the outcome given the exposure and the confounders.

### Genetic predictors of glycemic traits

We selected genetic instruments that were genome-wide significant (*p* < 5 × 10^−8^) and uncorrelated (*r*^*2*^ < 0.001) for fasting glucose (mmol/L), 2-h glucose (mmol/L), fasting insulin (natural log transformed pmol/L), and HbA_1c_ (%) from the Meta-Analyses of Glucose and Insulin-related traits Consortium (MAGIC), using data only from participants of European ancestry (*n* = 200,622)^12^. In this GWAS, participants with either type 1 or type 2 diabetes, who reported taking diabetes-related medications, had a fasting glucose ≥7 mmol/L, 2-h glucose ≥11.1 mmol/L, or HbA_1c_ ≥ 6.5% were excluded^12^. Genetic associations of glycemic traits were obtained using multivariable linear regression adjusted for age, sex, body-mass index (BMI) (except for HbA_1c_), study-specific covariates, and genomic control. The impact of collider bias due to BMI adjustment was minimal according to the original GWAS^12,13^.

### Genetic predictors of type 2 diabetes

We selected genetic instruments that were genome-wide significant (*p* < 5 × 10^−8^) and uncorrelated (*r*^*2*^ < 0.001) related to liability to type 2 diabetes from the largest GWAS to-date, using data only from participants of European ancestry (148,726 cases, 965,732 controls)^14^. Type 2 diabetes cases were ascertained using study-specific criteria, including diagnosis codes, hospital admission records, biochemical results (fasting glucose ≥ 7.0 mmol/L, or 2-h glucose ≥11.1 mmol/L, or HbA_1c_ ≥ 6.5%), and use of diabetes-related medications^14^. The genetic associations with type 2 diabetes were obtained using multivariable logistic regression adjusted for age, sex, and the top 10 principal components for genetic ancestry. Given type 2 diabetes is a binary variable, the interpretation of the corresponding Mendelian randomization study estimates using these instruments should be in terms of liability to type 2 diabetes^15^, consistent with earlier Mendelian randomization studies using diseases as the exposure^16–18^.

### Genetic associations with metabolomic markers

We obtained genetic associations with circulating metabolomic markers (*n* = 115,078) from UK Biobank summary statistics, accessed via MR-Base^19^. In brief, a range of circulating metabolomic markers were quantified in non-fasting EDTA-plasma samples collected from a random subset of the UK Biobank participants using a high-throughput nuclear magnetic resonance (NMR) spectroscopy platform from Nightingale Health, the technical details of which have been published^20^. We considered all 167 metabolomic biomarkers measured as outcomes, including amino acids, lipids, apolipoproteins, and lipoprotein subclass distribution and excluding glucose (Supplementary Data 1). Measurements of all metabolomic markers were inverse rank-normalized. Genetic associations with each biomarker (in standard deviations) were obtained using a linear mixed model with a random effect accounting for potential confounding due to population stratification and genetic relatedness, with adjustment for age, sex, fasting status, and genotyping array^21,22^. Whenever genetic instruments for glycemic traits and liability to type 2 diabetes were not available, proxy instruments (pairwise *r*^*2*^ ≥ 0.8) were identified based on the 1000 Genomes Phase 3 dataset (version 5, CEU reference population)^23^.

### Statistical analyses

We aligned the effect estimates for both exposure and outcome studies so that they corresponded to the same effect allele. Given that palindromic instruments (G > C and A > T) have the same allele notation on both the forward and reverse strand, we additionally used effect allele frequency (EAF) to ensure the alleles in both studies referred to the same strand direction. However, variants with ambiguous EAF (0.42 < EAF < 0.58) were excluded from the analyses. For each genetic instrument, we calculated the instrument strength for the Cragg-Donald F statistic using F = $\frac{{{{{{\rm{N}}}}}}-{{{{{\rm{K}}}}}}-1}{{{{{{\rm{K}}}}}}}\frac{{{{{{{\rm{R}}}}}}}^{2}}{1-{{{{{{\rm{R}}}}}}}^{2}}$, with an F statistic >10 indicating weak instrument bias is unlikely^24,25^. We also calculated the variance of each glycemic trait explained by the selected instruments (R^2^) based on F statistics, effect estimates, standard errors of the instruments, and the corresponding GWAS sample size^26,27^. We assessed the association of each glycemic trait and liability to type 2 diabetes with each circulating metabolite using inverse variance weighted with multiplicative random effects, which assumes no unbalanced horizontal pleiotropy^28^.

### Sensitivity analyses

Sensitivity analyses were conducted to assess the robustness of the results, which involved estimators relying on different assumptions. These included the weighted median, which requires at least 50% of the weighted estimates to be derived from valid instruments^29^, and MR-Egger, which allows all instruments to be pleiotropic as long as the instrument strength is independent of the direct effect, at the expense of a lower statistical power^30^. We also used the MR-Egger intercept term to explore the presence of unbalanced horizontal pleiotropy, where a statistically significant (*p* < 0.05) intercept implies its presence^30^. As shown in a previous GWAS, genetic instruments related to HbA_1c_ displayed possible horizontal pleiotropic effects via hemoglobin^12^. Therefore, we conducted multivariable Mendelian randomization (MVMR) for HbA_1c_-related analyses to control for horizontal pleiotropy by adjusting for hemoglobin concentration^31^. Genetic associations with hemoglobin concentration were obtained from the largest GWAS to date, which pooled data from the Blood Cell Consortium and UK Biobank and included 563,946 participants of European ancestry^32^. The genetic associations with hemoglobin concentration were obtained using multivariable linear regression adjusted for age, sex, principal components for genetic ancestry, and cohort specific-covariates^32^. The effect allele of each genetic variant was aligned to that for HbA_1c_^19^. We reported estimates from both MVMR- inverse variance weighted and MVMR-MR-Egger because the latter is robust to directional pleiotropy^33^. Horizontal pleiotropy was assessed using the MVMR-MR-Egger intercept.

### Reverse Mendelian randomization

To assess possible reverse causation, we conducted a reverse Mendelian randomization study to assess the association of NMR measured metabolomics (*n* = 157) on type 2 diabetes risk and glycemic traits. However, we did not include the panel on amino acids (*n* = 10) given this overlaps with ongoing and published work^34–36^.

### Statistics and reproducibility

All statistical analyses were conducted using R version 4.0.5, with the “TwoSampleMR”^19^ package used for data harmonization, extraction and alignment, univariable Mendelian randomization analyses, and the “MendelianRandomization”^37^ package used for MVMR. We calculated the number of principal components that explained 99% of the variance of the 167 metabolic measures using individual-level data from the UK Biobank (Application 14684), which gave 25 principal components. Given that 5 exposures were considered in this study, the threshold for statistical significance was set at *p* < 0.0004 (0.05/25/5).

### Ethics approval

This study only used publicly available summary statistics from relevant GWAS and UK Biobank, thus no ethics approval is required. Respective ethics approvals have been obtained by the GWAS investigator from all participating studies and the UK Biobank investigators from the North West Multi-center Research Ethic Committee.

### Reporting summary

Further information on research design is available in the Nature Portfolio Reporting Summary linked to this article.

## Results

### Genetic predictors for glycemic traits and liability to type 2 diabetes

We selected 67 genetic instruments for fasting glucose (R^2^: 4.7%, F statistics: 25 to 1662), 14 genetic instruments for 2-h glucose (R^2^: 1.3%, F statistics: 26 to 111), 74 genetic instruments for HbA_1c_ (R^2^: 5.6%, F statistics: 25 to 1392), 38 genetic instruments for fasting insulin (R^2^: 1.4%, F statistics: 22 to 173), and 228 instruments for liability to type 2 diabetes (F statistics: 29 to 3136) (Supplementary Data 2). Based on the F statistics, there was little evidence of weak instrument bias. Supplementary Fig. 1 shows the process on instrument selection.

### The association of fasting glucose and 2-h glucose with circulating metabolites

**Fig. 1:**
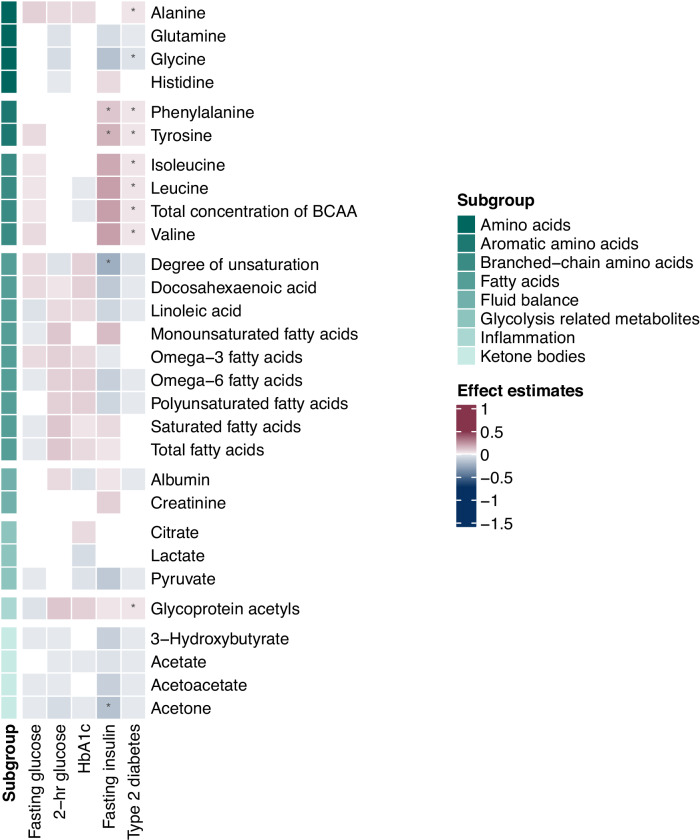
Heatmap of associations of genetically predicted glycemic traits (fasting glucose, 2-h glucose, HbA1c and fasting insulin) and of genetic liability to type 2 diabetes with amino acids, fatty acids, and various low-molecular weight metabolites. Circulating metabolites include: amino acids, aromatic amino acids, branched-chain amino acids, fatty acids, biomarkers of fluid balance, glycolysis related metabolites, inflammation, and ketone bodies. The estimates were obtained from Mendelian randomization analyses using the inverse variance weighted method. Asterisks depict statistical significance (*p* < 0.0004). BCAA branched-chain amino acid.

**Fig. 2:**
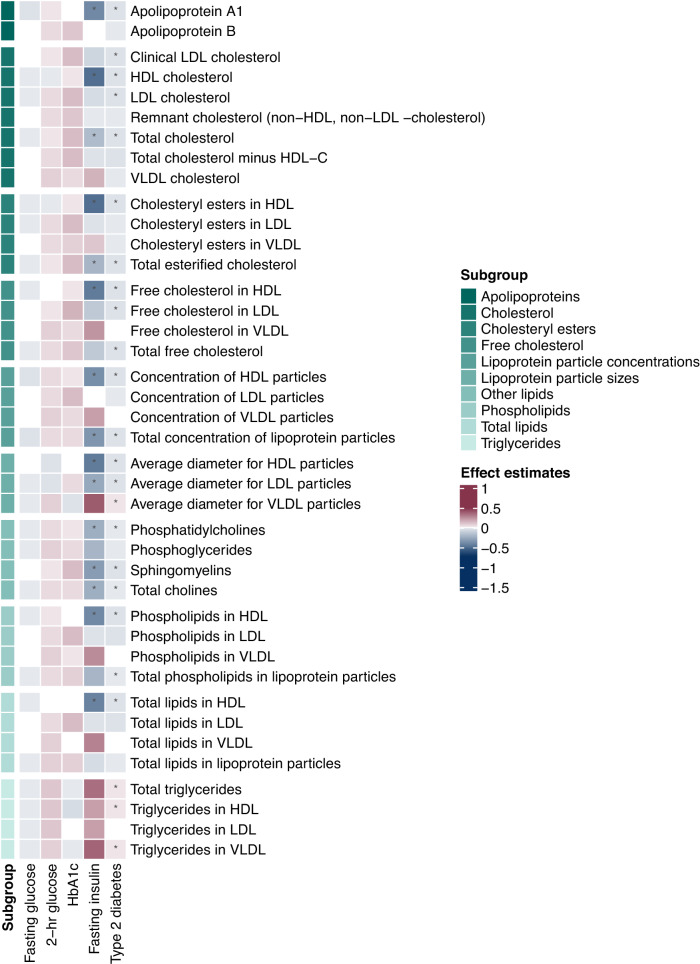
Heatmap of associations of genetically predicted glycemic traits (fasting glucose, 2-h glucose, HbA_1c_ and fasting insulin) and of genetic liability to type 2 diabetes with cholesterol metabolites. Measures of cholesterol metabolites include: apolipoproteins, cholesterol, cholesteryl esters, free cholesterol, lipoprotein particle concentrations, lipoprotein particle sizes, other lipids, phospholipids, total lipids, and triglycerides. The estimates were obtained from Mendelian randomization analyses using the inverse variance weighted method. Asterisks depict statistical significance (*p* < 0.0004). LDL low-density lipoprotein, HDL high-density lipoprotein, VLDL very low-density lipoprotein.

**Fig. 3:**
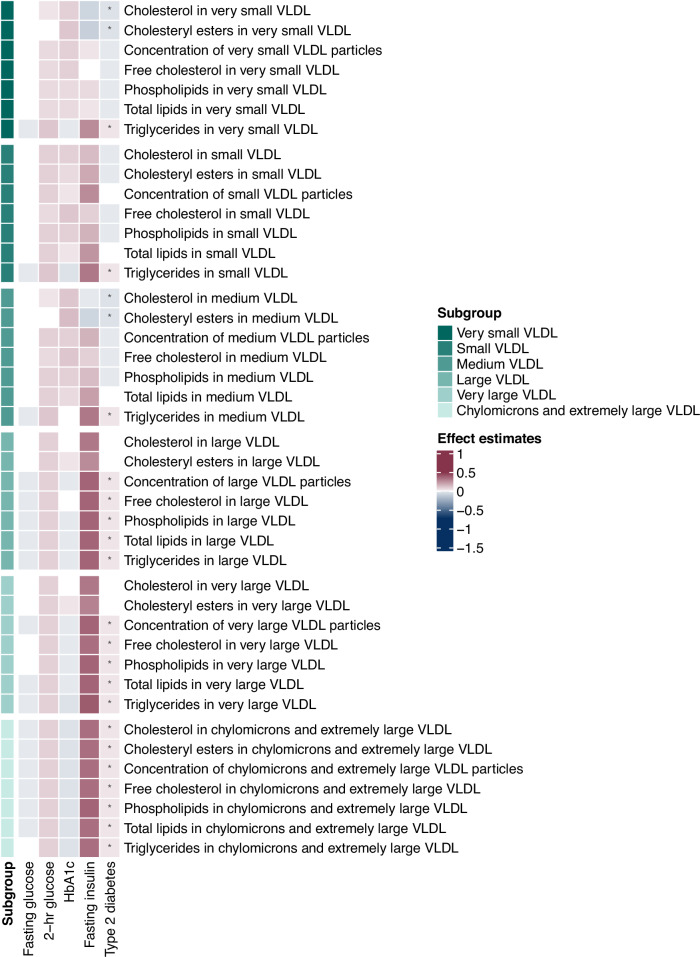
Heatmap of associations of genetically predicted glycemic traits (fasting glucose, 2-h glucose, HbA_1c_ and fasting insulin) and of genetic liability to type 2 diabetes with lipoprotein subclasses in very small, small, medium, large, very large VLDL, and chylomicrons and extremely large VLDL. The estimates were obtained from Mendelian randomization analyses using the inverse variance weighted method. Asterisks depict statistical significance (*p* < 0.0004). VLDL very low-density lipoprotein.

**Fig. 4:**
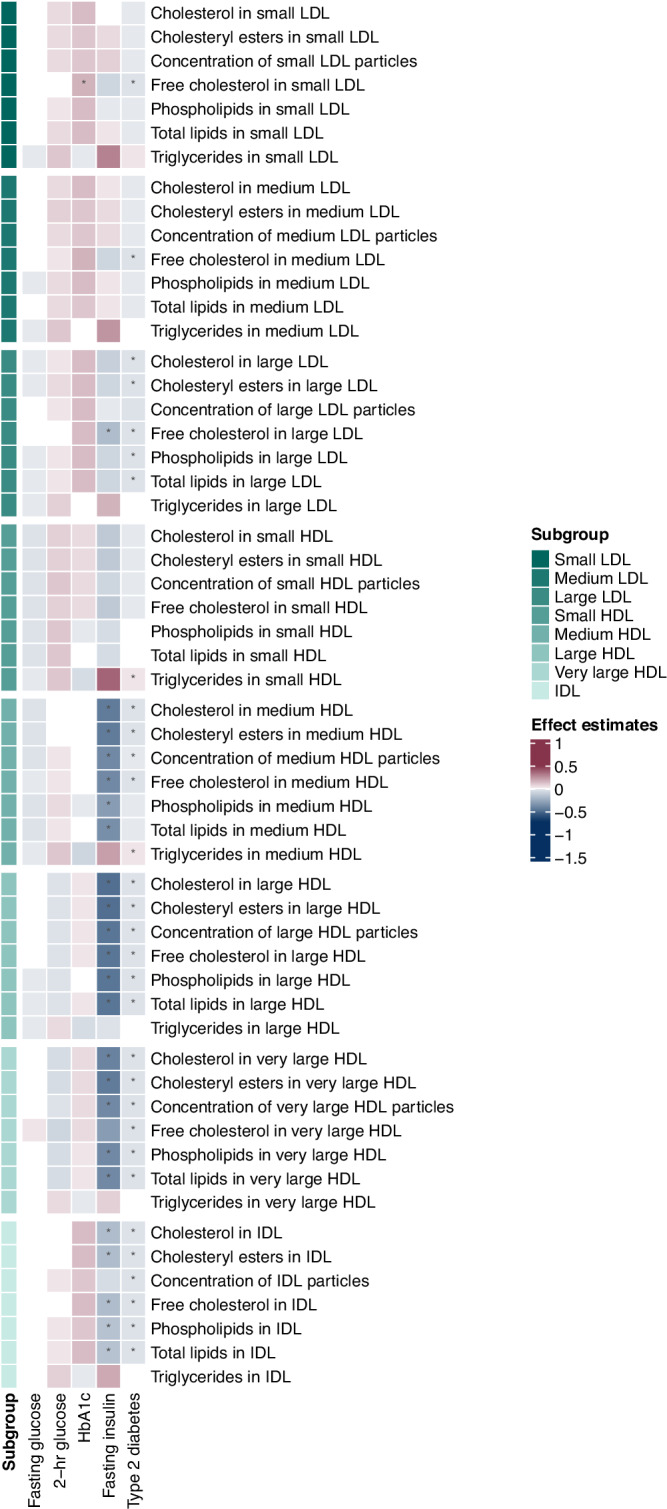
Heatmap of associations of genetically predicted glycemic traits (fasting glucose, 2-h glucose, HbA_1c_ and fasting insulin) and of genetic liability to type 2 diabetes with lipoprotein subfractions in small, medium, and large LDL, in small, medium, large, and very large HDL, and those in IDL. The estimates were obtained from Mendelian randomization analyses using the inverse variance weighted method. Asterisks depict statistical significance (*p* < 0.0004). LDL low-density lipoprotein, HDL high-density lipoprotein, IDL intermediate-density lipoprotein, VLDL very low-density lipoprotein.

Associations of all exposures with circulating metabolites are presented in Figs. 1–4. Results for fasting glucose and 2-h glucose are shown in Supplementary Data 3 and 4. None of the associations of these two glycemic traits with any of the metabolites achieved statistical significance.

### The association of HbA_1c_ with circulating metabolites

Higher HbA_1c_ was associated with higher free cholesterol in small LDL (Fig. 4). Estimates from both MR Egger and weighted median were directionally consistent. No evidence of horizontal pleiotropy was observed (Supplementary Data 5). After adjusting for hemoglobin using MVMR, the positive association of HbA_1c_ with free cholesterol in small LDL was slightly attenuated (Supplementary Data 6).

### The association of fasting insulin with circulating metabolites

There were 42 associations of higher fasting insulin with circulating metabolites that achieved statistical significance, while only two had a positive effect estimate (Supplementary Data 7). Notably, higher fasting insulin was associated with higher aromatic amino acids (phenylalanine and tyrosine) (Fig. 1). Higher fasting insulin was associated with lower apolipoprotein A1, total cholesterol, HDL-cholesterol and other lipid components in HDL, and total cholines (Fig. 2). There was also a consistent inverse association of fasting insulin with lipoprotein subfractions in medium HDL, large HDL, and very large HDL, as well as IDL (except for triglycerides) (Fig. 4). No evidence of horizontal pleiotropy was observed for any of these associations (Supplementary Data 7).

### The association of liability to type 2 diabetes with circulating metabolites

There were 88 associations of type 2 diabetes with circulating metabolites, comprising 34 positive and 54 inverse associations (Supplementary Data 8). The metabolomic signature of type 2 diabetes included most fasting insulin-associated signals (38 out of 42), except for degree of unsaturation in fatty acids, acetone (Fig. 1), phospholipids in medium HDL, and total lipids in medium HDL (Fig. 4). Besides aromatic amino acids, type 2 diabetes was also associated with higher BCAAs (isoleucine, leucine, valine and total BCAA), amino acid (alanine) and glycoprotein acetyls (Fig. 1). Moreover, type 2 diabetes showed positive associations with total triglycerides, triglycerides in HDL, and triglycerides in VLDL (Fig. 2). Consistently, positive associations of type 2 diabetes with lipoprotein subfractions in large VLDL, very large VLDL, and chylomicrons and extremely large VLDL were found (Fig. 3). There was a consistent inverse association of type 2 diabetes with lipoprotein subfractions in large LDL, medium HDL, large HDL, very large HDL and IDL (except triglycerides) (Fig. 4).

### The association of circulating metabolites with glycemic traits and T2D risk

In the reverse Mendelian randomization analyses (Supplementary Data 9–13), there were no associations of circulating metabolites with fasting glucose and 2-h glucose (Supplementary Figs. 2–4). Linoleic acid was associated with lower HbA_1c_ (Supplementary Fig. 2). However, HDL-cholesterol and cholesteryl ester in HDL were inversely associated with fasting insulin, suggesting potential reverse causation (Supplementary Fig. 3). There were 19 circulating metabolites associated with lower type 2 diabetes risk, of which 10 overlapped with the associations of type 2 diabetes with metabolites, such as LDL-cholesterol and subfractions (cholesterol, cholesteryl ester, free cholesterol, phospholipids, total lipids) in large LDL (Supplementary Data 13).

## Discussion

This Mendelian randomization studies explored the metabolomic signatures of different glycemic traits and liability to type 2 diabetes. Specifically, fasting glucose and 2-h glucose did not impact lipid profile, consistent with a previous Mendelian randomization study but not other observations studies^9,38,39^, although these studies did not investigate 2-h glucose. HbA_1c_ was positively associated with LDL-cholesterol, as well as with free cholesterol and phospholipids in LDL, which were consistent with a previous Mendelian randomization study conducted in Chinese (*n* = 11,935)^40^ and other observational studies^41^. Although these glycemic traits were broadly used in defining type 2 diabetes, we found that liability to type 2 diabetes had a strikingly different metabolomic signature compared to fasting glucose and 2-h glucose, and HbA_1c_, such as higher BCAAs, aromatic amino acids, alanine, and lower lipoprotein subfractions, which has been reported in a previous Mendelian randomization study^38^. Our study adds by showing that metabolomic signatures associated with liability to type 2 diabetes resemble the signatures for fasting insulin, which implies that signals of liability to type 2 diabetes cannot be solely explained by hyperglycemia but is likely more related to the consequence of elevated insulin.

As with all study designs, the validity of Mendelian randomization studies depends on assumptions^42^. Whilst Mendelian randomization studies are less susceptible to confounding due to the use of genetics randomly allocated at conception and weak instrument bias was unlikely given the high F statistics, there could be issues with violation of exclusion restriction assumptions where there were signs of horizontal pleiotropy for some analyses. However, the results of sensitivity analyses, which utilized estimators based on different sets of assumptions for validity, gave similar conclusions and hence the associations were unlikely driven completely by the violation of assumptions. We acknowledge that 37.5% of the participants in the GWAS of type 2 diabetes also provided data for the outcome GWAS but biases arising from using two-sample Mendelian randomization methods were likely little given the large F statistics for the instruments. For MR-Egger, the high instrument variability (I^2^_GX_ for all included instruments of type 2 diabetes was 0.99) indicated that biases in MR-Egger estimates due to sample overlap are likely minimal^43,44^. Furthermore, we were not able to explore the effect of type 2 diabetes on metabolic signatures since we only instrumented on liability to type 2 diabetes. As such, results from liability to type 2 diabetes should be interpreted with caution^15^, where the results could be a mixture of both causes, consequences and merely biomarkers related to type 2 diabetes in the general population with some diagnosed with type 2 diabetes^45^. Despite these limitations, Mendelian randomization studies in general give findings more consistent with randomized controlled trials than conventional observational studies^46^.

Previous observational studies showed positive associations of fasting glucose with BCAAs^9^, cholesterols in VLDLs, or saturated and unsaturated fatty acids, as well as with lower phospholipids and sphingomyelins^39^. However, confounding cannot be ruled out completely, especially as these studies had limited sample sizes which impacted the ability to adjust for confounders. Furthermore, reverse causation could be an issue, where prospective cohort studies showed these metabolomic markers associated with higher risk of impaired fasting glucose^47^. The lack of association in our study supported that these previous observations were likely non-causal. Our study is also explored the association of 2-h glucose with metabolomic signature, which showed null associations with included metabolomic signatures.

HbA_1c_ is often used to proxy average glucose over 2–3 months. As these associations were not observed for fasting glucose, these may reflect differences in hyperglycemia being proxied by these two glycemic traits or glycemia-independent effects of HbA_1c_. One possibility is hemoglobin^48^, which HbA_1c_ is strongly linked to and may also impact lipid levels, although these associations remained after adjusting for hemoglobin using MVMR^49^. Similarly, other diseases such as glucose-6-phosphate dehydrogenase deficiency and changes in iron homeostasis markers may also reduce HbA_1c_^50^. Better understanding of the underlying mechanisms has substantial implications for understanding the impact of hyperglycemia, as proxied by fasting glucose, 2-h glucose or HbA_1c_.

The similarities in metabolic signature between liability to type 2 diabetes and fasting insulin, but not other glycemic traits, imply signals associated with type 2 diabetes liability are likely the result of elevated insulin in response to insulin resistance instead of overall hyperglycemia. The lipid signatures, such as inverse associations with IDL and HDL and potentially positive association with VLDL, were similar to a previous smaller Mendelian randomization although that study only focused on liability to type 2 diabetes^51^. Our finding concerning insulin is also consistent with a previous study showing insulin resistance associated with higher BCAAs^52^, possibly via decreased BCAA metabolism due to impaired insulin action^53^. Previous studies have debated whether BCAA is a cause of insulin resistance^53^, although previous Mendelian randomization studies suggested a non-causal role of BCAAs in insulin resistance^54^. Additional mechanistic studies would be useful to identify the different roles of BCAAs, insulin, and type 2 diabetes and hence targets of intervention.

Although this study used Mendelian randomization, which is less susceptible to confounding, we caution readers with several limitations when interpreting the findings. First, valid causal inference depends on satisfying the instrumental variable assumptions when fully assessing pleiotropy is challenging^55^. Nonetheless, several sensitivity analyses with different assumptions about pleiotropy yielded consistent conclusions. Second, since this study utilized data from European populations only, our findings may not generalize to other populations. Assessing metabolic signatures across ethnicities would be helpful, when suitable GWAS becomes available. Third, there were signs of reverse causation for lipids related traits after correcting for multiple comparisons, which were consistent with previous Mendelian randomization studies^56,57^. However, these results should be interpreted with caution, in particular the instruments used for NMR measured traits could be pleiotropic, where correction of horizontal pleiotropy may not be adequate using standard sensitivity analyses. Based on the findings from this study, future studies with more specific hypotheses (e.g., focusing on specific lipid phenotype), coupled with the use of MVMR which could better account for horizontal pleiotropy via other lipids, and the use of larger lipid GWAS^58^, will help ascertain the findings from our reverse Mendelian randomization study^2,59,60^. Fourth, genetic instruments for glycemic traits were obtained from MAGIC, which excluded participants with type 2 diabetes. Although such an approach should reduce the likelihood of reverse causation, this may inevitably introduce possible selection bias^42^. Lastly, the metabolomic markers used in this study were mainly related to lipids although other markers relevant to type 2 diabetes, such as BCAAs, were also included. Whether there are shared signatures in other metabolomic markers across glycemic traits require further investigation.

In conclusion, fasting glucose, 2-h glucose, and HbA_1c_ had little evidence of a metabolomic signature for the metabolites considered. Fasting insulin and liability to type 2 diabetes had similar metabolic signatures encompassing a wide range of lipids and amino acids. As such, glycemic traits likely reflect symptoms of type 2 diabetes while insulin also has a role in the pathophysiology of type 2 diabetes independent of hyperglycemia. The distinct characterization of these inter-related glycemic traits may help us better understand the mechanisms underpinning the relation of these traits with downstream clinical outcomes such as cardiovascular diseases, as well as diagnosis and clinical management of type 2 diabetes using these traits.

### Supplementary information

Supplementary information

Description of Additional Supplementary Files

Supplementary Data 1-13

Reporting Summary

## Data Availability

Summary statistics of glycemic traits are available from the MAGIC, with details provided in Supplementary Data 2. Summary statistics of NMR metabolites are available from the IEU GWAS database (https://gwas.mrcieu.ac.uk/), with the IDs for each metabolite listed in Supplementary Data 1.
