# Supplementary material for: A two-sample Mendelian randomization study explores metabolic profiling of different glycemic traits
Source: Commun Biol. 2024 Mar 8;7:293. doi: 10.1038/s42003-024-05977-1 (PMC10923832; doi:10.1038/s42003-024-05977-1)
Supplement: Supplementary file 1 — Supplementary information [file 42003_2024_5977_MOESM1_ESM.pdf]

Supplementary Information

A two-sample Mendelian randomization study explores metabolic profiling of different glycemic traits

Tommy HT Wong,[a] Jacky MY Mo,[a] Mingqi Zhou, Jie V Zhao, C Mary Schooling, Baoting He, Shan Luo,[*] Shiu Lun Au Yeung

[a] Contributed equally

*Corresponding author

Index

Supplementary Figure 1. Flowchart for genetic instrument selection for glycemic traits (fasting glucose, 2-hour glucose, HbA_{1c} and fasting insulin) and liability to type 2 diabetes

Supplementary Figure 2. Heatmap of associations of genetically predicted circulating fatty acids and various low-molecular weight metabolites on glycemic traits (fasting glucose, 2-hour glucose, HbA_{1c} and fasting insulin) and type 2 diabetes risk

Supplementary Figure 3. Heatmap of associations of genetically predicted circulating cholesterol metabolites on glycemic traits (fasting glucose, 2-hour glucose, HbA_{1c} and fasting insulin) and type 2 diabetes risk

Supplementary Figure 4. Heatmap of associations of 14 genetically predicted lipoprotein subclasses on glycemic traits (fasting glucose, 2-hour glucose, f HbA_{1c} and fasting insulin) and type 2 diabetes risk

Supplementary Figure 1. Flowchart for genetic instrument selection for glycemic traits and liability to type 2 diabetes

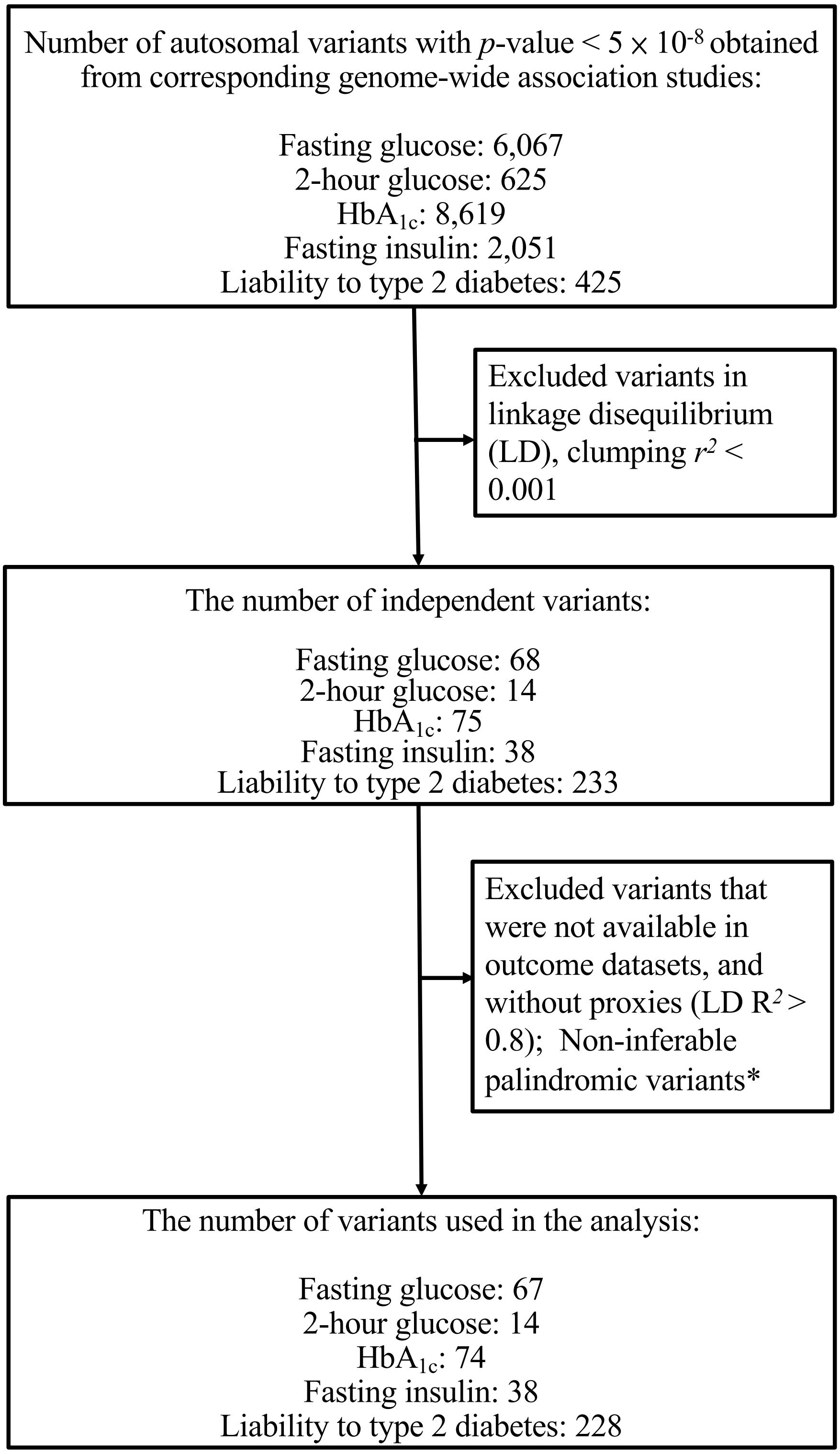

Non-inferable palindromic variants, with effect allele frequencies between 0.42 and 0.58.

Supplementary Figure 2. Heatmap of associations of genetically predicted circulating fatty acids and various low-molecular weight metabolites on glycemic traits (fasting glucose, 2-hour glucose, HbA_{1c} and fasting insulin) and type 2 diabetes risk

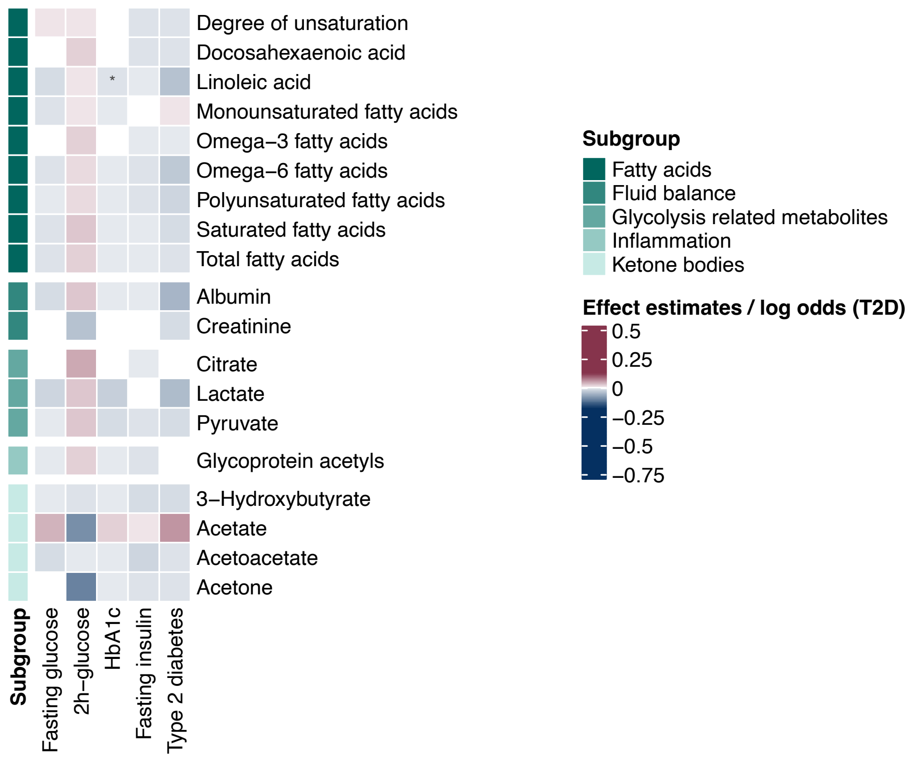

Circulating metabolites include: fatty acids, biomarkers of fluid balance, glycolysis related metabolites, inflammation, and ketone bodies. The estimates were obtained from Mendelian randomization analyses using the inverse variance weighted method. Asterisks depict statistical significance ($p < 0.0004$).

Supplementary Figure 3. Heatmap of associations of genetically predicted circulating cholesterol metabolites on glycemic traits (fasting glucose, 2-hour glucose, HbA_{1c} and fasting insulin) and type 2 diabetes risk

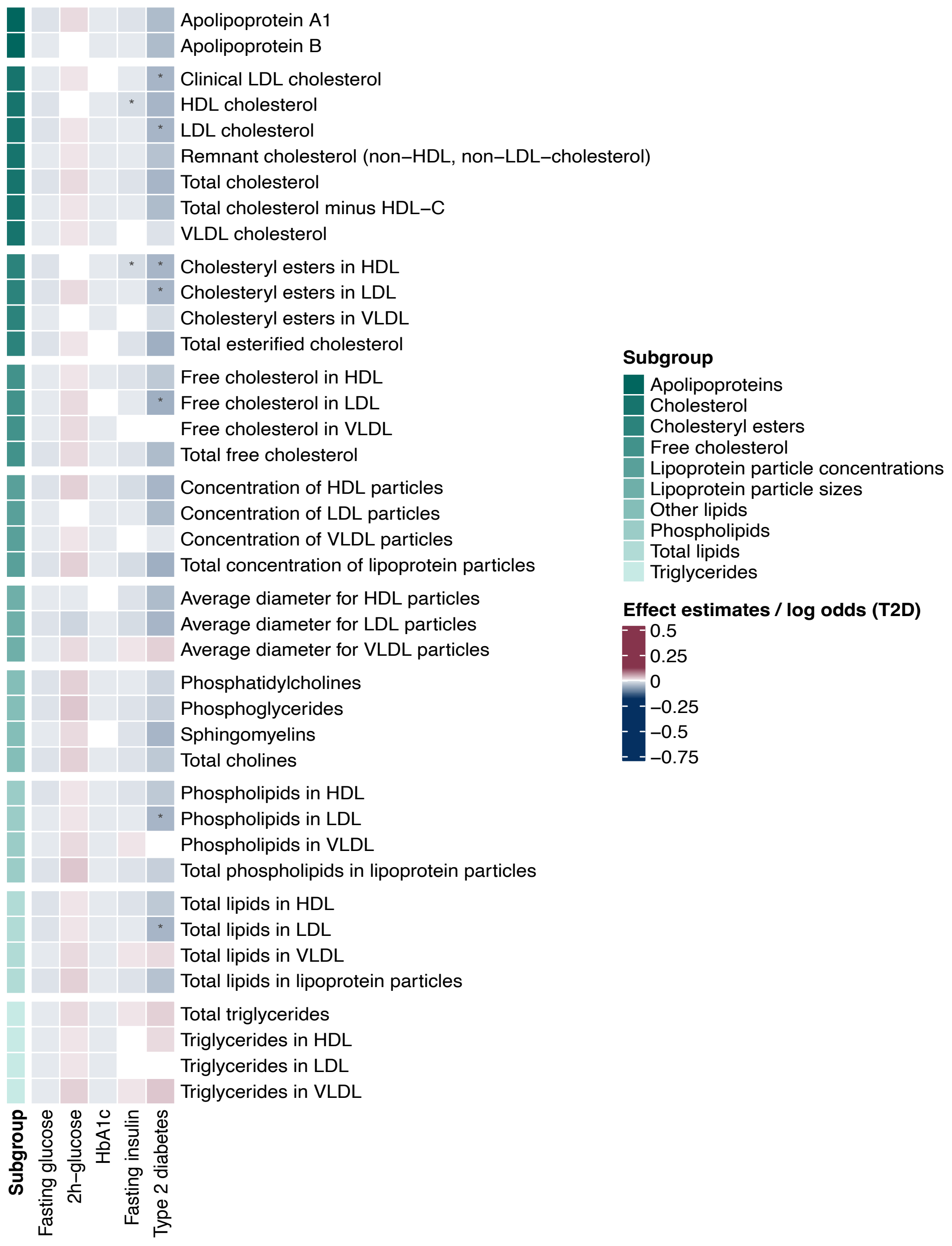

Measures of cholesterol metabolites include: apolipoproteins, cholesterol, cholesteryl esters, free cholesterol, lipoprotein particle concentrations, lipoprotein particle sizes, other lipids, phospholipids, total lipids, and triglycerides. The estimates were obtained from Mendelian randomization analyses using the inverse variance weighted method. Asterisks depict statistical significance ($p < 0.0004$). LDL, low-density lipoprotein. HDL, high-density lipoprotein. VLDL, very low-density lipoprotein.

Supplementary Figure 4. Heatmap of associations of 14 genetically predicted lipoprotein subclasses on glycemic traits (fasting glucose, 2-hour glucose, f HbA_{1c} and fasting insulin) and type 2 diabetes risk

a)

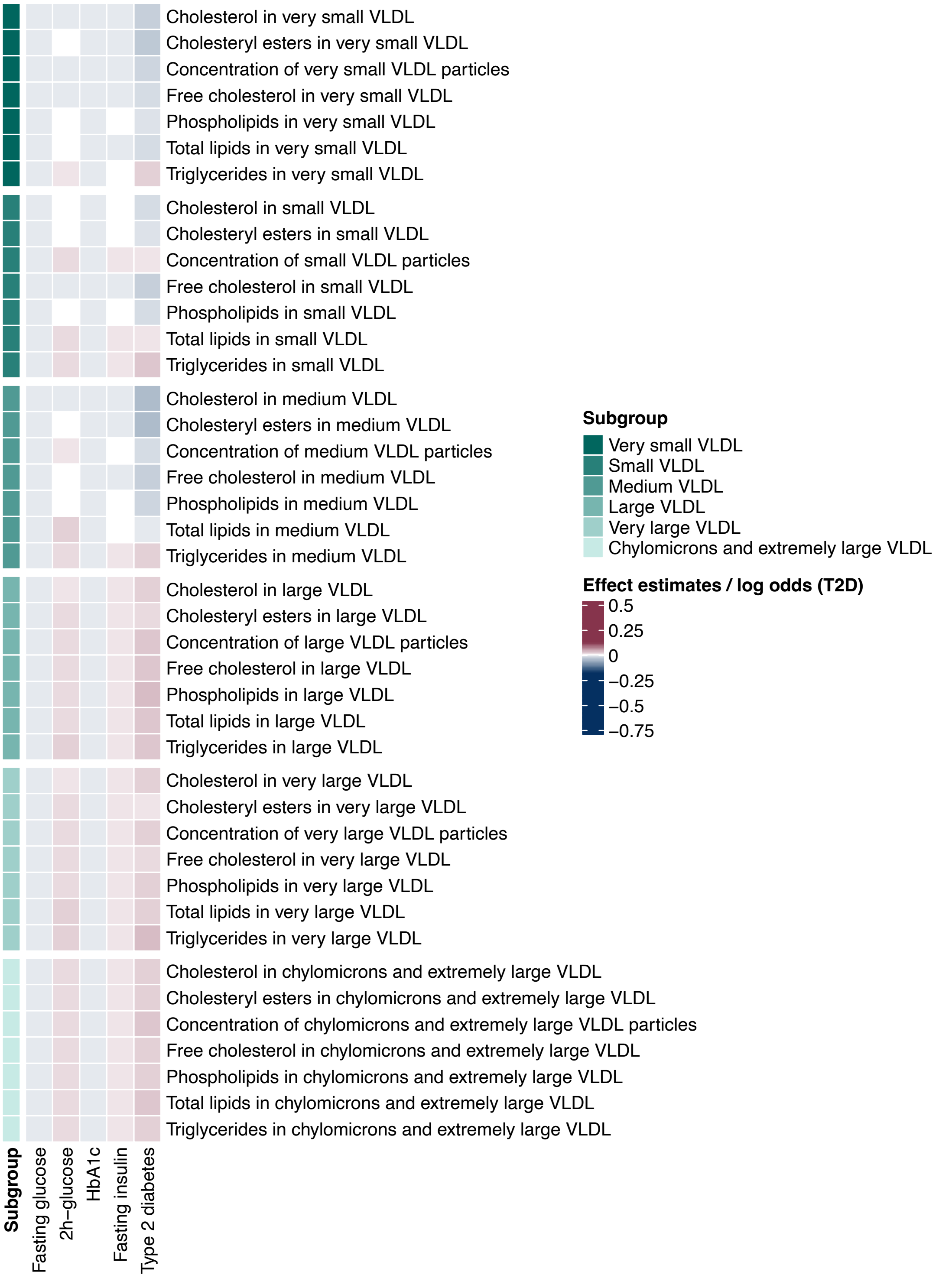

b)

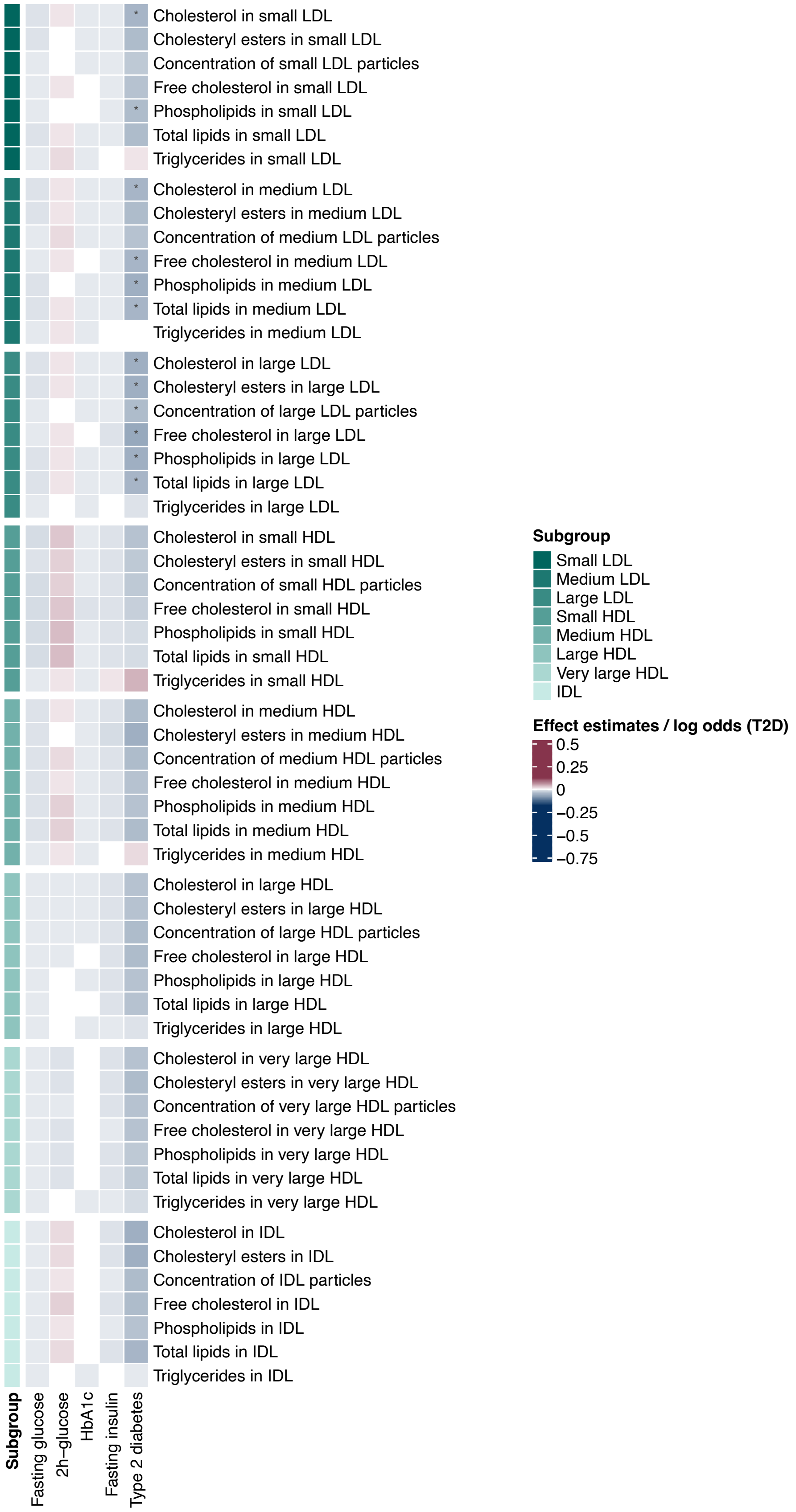

Cholesterol in small LDL
Cholesteryl esters in small LDL
Concentration of small LDL particles
Free cholesterol in small LDL
Phospholipids in small LDL
Total lipids in small LDL
Triglycerides in small LDL
Cholesterol in medium LDL
Cholesteryl esters in medium LDL
Concentration of medium LDL particles
Free cholesterol in medium LDL
Phospholipids in medium LDL
Total lipids in medium LDL
Triglycerides in medium LDL
Cholesterol in large LDL
Cholesteryl esters in large LDL
Concentration of large LDL particles
Free cholesterol in large LDL
Phospholipids in large LDL
Total lipids in large LDL
Triglycerides in large LDL
Cholesterol in small HDL
Cholesteryl esters in small HDL
Concentration of small HDL particles
Free cholesterol in small HDL
Phospholipids in small HDL
Total lipids in small HDL
Triglycerides in small HDL
Cholesterol in medium HDL
Cholesteryl esters in medium HDL
Concentration of medium HDL particles
Free cholesterol in medium HDL
Phospholipids in medium HDL
Total lipids in medium HDL
Triglycerides in medium HDL
Cholesterol in large HDL
Cholesteryl esters in large HDL
Concentration of large HDL particles
Free cholesterol in large HDL
Phospholipids in large HDL
Total lipids in large HDL
Triglycerides in large HDL
Cholesterol in very large HDL
Cholesteryl esters in very large HDL
Concentration of very large HDL particles
Free cholesterol in very large HDL
Phospholipids in very large HDL
Total lipids in very large HDL
Triglycerides in very large HDL
Cholesterol in IDL
Cholesteryl esters in IDL
Concentration of IDL particles
Free cholesterol in IDL
Phospholipids in IDL
Total lipids in IDL
Triglycerides in IDL
Subgroup
Fasting glucose
2h−glucose
HbA1c
Fasting insulin
Type 2 diabetes
Subgroup
Small LDL
Medium LDL
Large LDL
Small HDL
Medium HDL
Large HDL
Very large HDL
IDL
Effect estimates / log odds (T2D)
0.5
0.25
0
−0.25
−0.5
−0.75

Lipoprotein subclasses in panel a): lipoprotein subfractions in very small, small, medium, large, very large VLDL, and chylomicrons and extremely large VLDL; and panel b): lipoprotein subfractions in small, medium, and large LDL, in small, medium, large, and very large HDL and those in IDL. The estimates were obtained from Mendelian randomization analyses using the inverse variance weighted method. Asterisks depict statistical significance ($p < 0.0004$). HDL, high-density lipoprotein. IDL, intermediate-density lipoprotein. LDL, low-density lipoprotein. VLDL, very low-density lipoprotein.
