# Supplementary material for: A two-sample Mendelian randomization study explores metabolic profiling of different glycemic traits
Source: Commun Biol. 2024 Mar 8;7:293. doi: 10.1038/s42003-024-05977-1 (PMC10923832; doi:10.1038/s42003-024-05977-1)
Supplement: Supplementary file 2 — Description of Additional Supplementary Files [file 42003_2024_5977_MOESM2_ESM.pdf]

Description of Additional Supplementary Files

File name: Supplementary Data 1
Description: Circulating metabolites included as the outcomes in this study

File name: Supplementary Data 2
Description: Genetic instruments selected for each exposure

File name: Supplementary Data 3
Description: Effect of genetically predicted fasting glucose (FG, mmol/L) on 167 circulating metabolites (SD)

File name: Supplementary Data 4
Description: Effect of genetically predicted 2-hour glucose (2hGlu, mmol/L) on 167 circulating metabolites (SD)

File name: Supplementary Data 5
Description: Effect of genetically predicted glycated hemoglobin (HbA1c, %) on 167 circulating metabolites (SD)

File name: Supplementary Data 6
Description: Effect of genetically predicted glycated hemoglobin (HbA1c, %) on 167 circulating metabolites (SD) after adjusted for hemoglobin level

File name: Supplementary Data 7
Description: Effect of genetically predicted fasting insulin (FI, log transformed pmol/L) on 167 circulating metabolites (SD)

File name: Supplementary Data 8
Description: Effect of genetic liability to type 2 diabetes (log odds) on 167 circulating metabolites (SD)

File name: Supplementary Data 9
Description: Effect of genetically predicted circulating metabolites (SD) on fasting glucose (FG, mmol/L)

File name: Supplementary Data 10
Description: Effect of genetically predicted circulating metabolites (SD) on 2-hour glucose (2hGlu, mmol/L)

File name: Supplementary Data 11
Description: Effect of genetically predicted circulating metabolites (SD) on glycated hemoglobin (HbA1c, %)

File name: Supplementary Data 12
Description: Effect of genetically predicted circulating metabolites (SD) on fasting insulin (FI, log transformed pmol/L)

File name: Supplementary Data 13
Description: Effect of genetically predicted circulating metabolites (SD) on type 2 diabetes risk (log odds, DIAMANTE, Mahajan 2018, no UKB)
