# Supplementary material for: A two-sample Mendelian randomization study explores metabolic profiling of different glycemic traits
Source: Commun Biol. 2024 Mar 8;7:293. doi: 10.1038/s42003-024-05977-1 (PMC10923832; doi:10.1038/s42003-024-05977-1)
Supplement: Supplementary file 4 — Reporting Summary [file 42003_2024_5977_MOESM4_ESM.pdf]

nature portfolio

Corresponding author(s): Shan Luo

Last updated by author(s): Jan 4, 2024

Reporting Summary

Nature Portfolio wishes to improve the reproducibility of the work that we publish. This form provides structure for consistency and transparency in reporting. For further information on Nature Portfolio policies, see our Editorial Policies and the Editorial Policy Checklist.

Statistics

For all statistical analyses, confirm that the following items are present in the figure legend, table legend, main text, or Methods section.

n/a	Confirmed	
☐	☒	The exact sample size (*n*) for each experimental group/condition, given as a discrete number and unit of measurement
☐	☒	A statement on whether measurements were taken from distinct samples or whether the same sample was measured repeatedly
☐	☒	The statistical test(s) used AND whether they are one- or two-sided<br>*Only common tests should be described solely by name; describe more complex techniques in the Methods section.*
☐	☒	A description of all covariates tested
☐	☒	A description of any assumptions or corrections, such as tests of normality and adjustment for multiple comparisons
☐	☒	A full description of the statistical parameters including central tendency (e.g. means) or other basic estimates (e.g. regression coefficient) AND variation (e.g. standard deviation) or associated estimates of uncertainty (e.g. confidence intervals)
☐	☒	For null hypothesis testing, the test statistic (e.g. *F*, *t*, *r*) with confidence intervals, effect sizes, degrees of freedom and *P* value noted<br>*Give P values as exact values whenever suitable.*
☒	☐	For Bayesian analysis, information on the choice of priors and Markov chain Monte Carlo settings
☒	☐	For hierarchical and complex designs, identification of the appropriate level for tests and full reporting of outcomes
☐	☒	Estimates of effect sizes (e.g. Cohen's *d*, Pearson's *r*), indicating how they were calculated

Our web collection on statistics for biologists contains articles on many of the points above.

Software and code

Policy information about availability of computer code

Data collection	n/a
Data analysis	R (version 4.0.5) and R packages "TwoSampleMR" and "MendelianRandomization"

For manuscripts utilizing custom algorithms or software that are central to the research but not yet described in published literature, software must be made available to editors and reviewers. We strongly encourage code deposition in a community repository (e.g. GitHub). See the Nature Portfolio guidelines for submitting code & software for further information.

Data

Policy information about availability of data

All manuscripts must include a data availability statement. This statement should provide the following information, where applicable:

- Accession codes, unique identifiers, or web links for publicly available datasets
- A description of any restrictions on data availability
- For clinical datasets or third party data, please ensure that the statement adheres to our policy

Summary statistics of glycemic traits are available from the MAGIC (https://magicinvestigators.org/). Summary statistics of NMR metabolites are available from the IEU GWAS database (https://gwas.mrcieu.ac.uk/).

Research involving human participants, their data, or biological material

Policy information about studies with human participants or human data. See also policy information about sex, gender (identity/presentation), and sexual orientation and race, ethnicity and racism.

Reporting on sex and gender	To maximize the statistical power in this two-sample Mendelian randomization, we did not consider sex-specific effects of exposures on the outcomes.
Reporting on race, ethnicity, or other socially relevant groupings	To minimize potential bias from population stratification, we only used data from participants of European ancestry.
Population characteristics	See below.
Recruitment	n/a
Ethics oversight	This study only used publicly available summary statistics from relevant genome-wide association studies and the UK Biobank, thus no ethics approval is required. Respective ethics approvals have been obtained by the genome-wide association studies and the UK Biobank investigators.

Note that full information on the approval of the study protocol must also be provided in the manuscript.

Field-specific reporting

Please select the one below that is the best fit for your research. If you are not sure, read the appropriate sections before making your selection.

☒ Life sciences ☐ Behavioural & social sciences ☐ Ecological, evolutionary & environmental sciences

For a reference copy of the document with all sections, see nature.com/documents/nr-reporting-summary-flat.pdf

Life sciences study design

All studies must disclose on these points even when the disclosure is negative.

Sample size	Summary statistics of fasting glucose (mmol/L), 2-hour glucose (mmol/L), fasting insulin (natural log transformed pmol/L), and HbA1c (%) from the Meta-Analyses of Glucose and Insulin-related traits Consortium (MAGIC) from participants of European ancestry (n=200,622); type 2 diabetes (148,726 cases, 965,732 controls) from the largest genome-wide association study; and 167 circulating metabolomic bio-markers (n=115,078) from the UK Biobank.
Data exclusions	In MAGIC, the participants with either type 1 or type 2 diabetes, who reported taking diabetes-related medications, had a fasting glucose ≥ 7 mmol/L, 2-hour glucose ≥ 11.1 mmol/L, or HbA1c ≥ 6.5% were excluded.
Replication	The results were not replicated in another dataset, as we have used the largest publicly available data to-dated with sufficient sample size for this two-sample Mendelian randomization study.
Randomization	Mendelian randomization study designs use genetic variants assigned randomly at conception, which is analogous to randomization in randomized controlled trials.
Blinding	n/a

Reporting for specific materials, systems and methods

We require information from authors about some types of materials, experimental systems and methods used in many studies. Here, indicate whether each material, system or method listed is relevant to your study. If you are not sure if a list item applies to your research, read the appropriate section before selecting a response.

Materials & experimental systems

n/a	Involved in the study	
☒	☐	Antibodies
☒	☐	Eukaryotic cell lines
☒	☐	Palaeontology and archaeology
☒	☐	Animals and other organisms
☒	☐	Clinical data
☒	☐	Dual use research of concern
☒	☐	Plants

Methods

n/a	Involved in the study	
☒	☐	ChIP-seq
☒	☐	Flow cytometry
☒	☐	MRI-based neuroimaging

Plants

Seed stocks

Report on the source of all seed stocks or other plant material used. If applicable, state the seed stock centre and catalogue number. If plant specimens were collected from the field, describe the collection location, date and sampling procedures.

Novel plant genotypes

Describe the methods by which all novel plant genotypes were produced. This includes those generated by transgenic approaches, gene editing, chemical/radiation-based mutagenesis and hybridization. For transgenic lines, describe the transformation method, the number of independent lines analyzed and the generation upon which experiments were performed. For gene-edited lines, describe the editor used, the endogenous sequence targeted for editing, the targeting guide RNA sequence (if applicable) and how the editor was applied.

Authentication

Describe any authentication procedures for each seed stock used or novel genotype generated. Describe any experiments used to assess the effect of a mutation and, where applicable, how potential secondary effects (e.g. second site T-DNA insertions, mosiacism, off-target gene editing) were examined.
